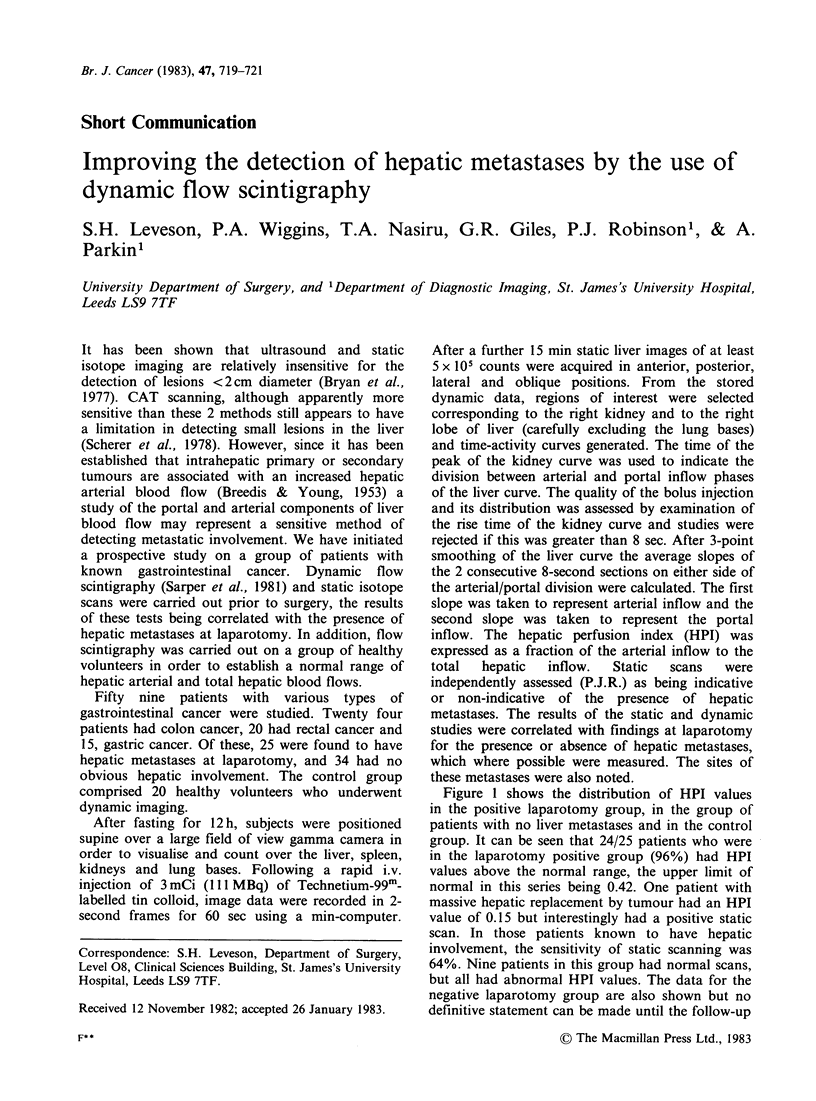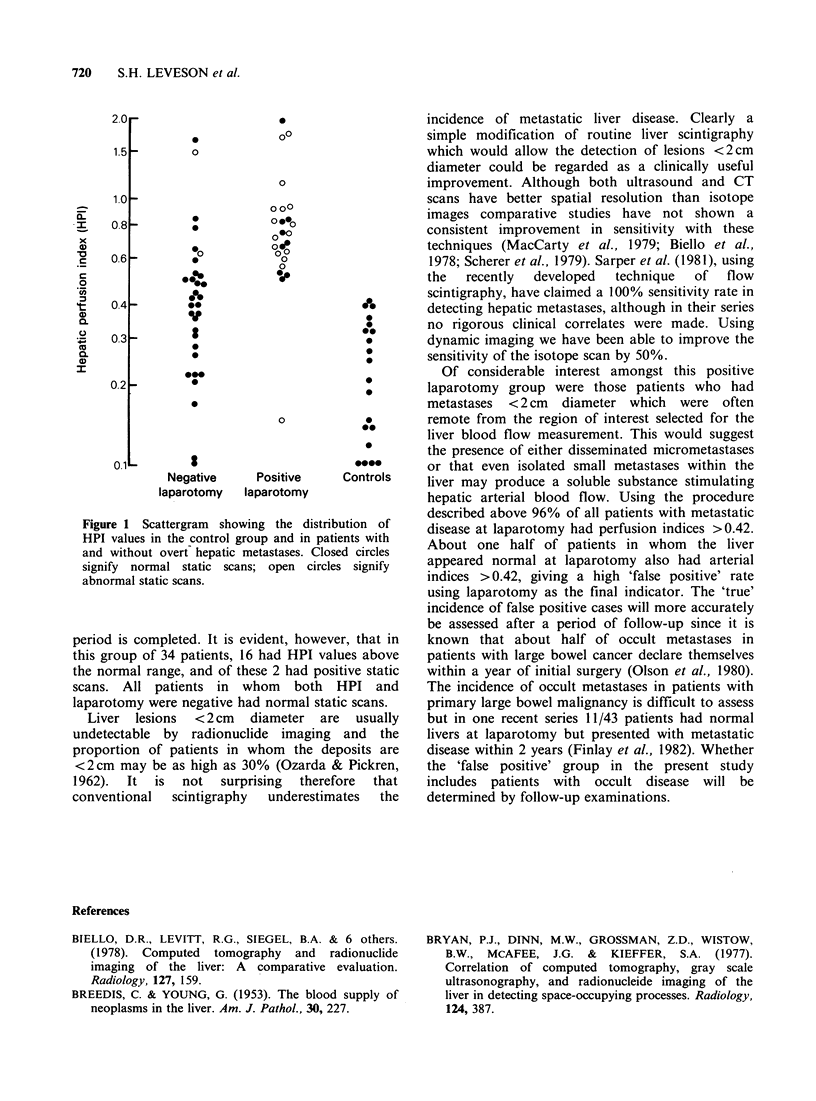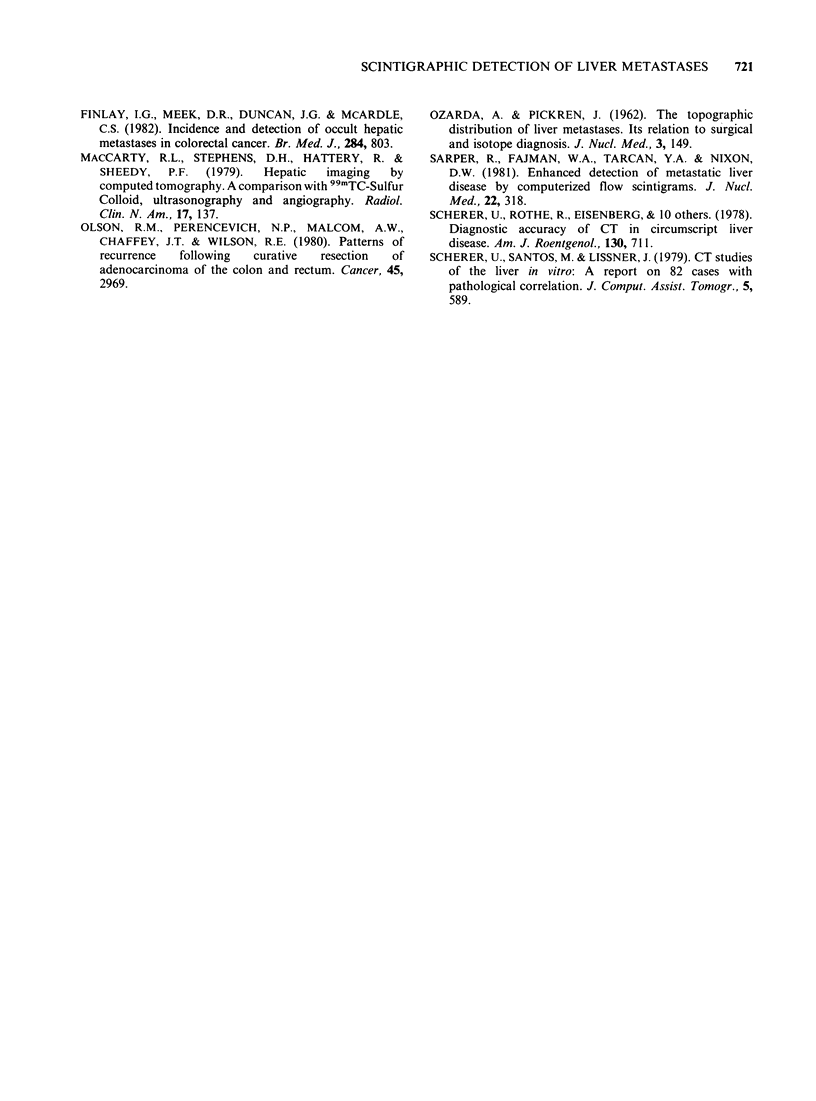# Improving the detection of hepatic metastases by the use of dynamic flow scintigraphy.

**DOI:** 10.1038/bjc.1983.111

**Published:** 1983-05

**Authors:** S. H. Leveson, P. A. Wiggins, T. A. Nasiru, G. R. Giles, P. J. Robinson, A. Parkin


*Br. J. Cancer* (1983), **47**, 719–721

### Short Communication

### Improving the detection of hepatic metastases by the use of dynamic flow scintigraphy

S.H. Leveson, P.A. Wiggins, T.A. Nasiru, G.R. Giles, P.J. Robinson[1], & A. Parkin[1]

*University Department of Surgery, and [1]Department of Diagnostic Imaging, St. James's University Hospital, Leeds LS9 7TF*

It has been shown that ultrasound and static isotope imaging are relatively insensitive for the detection of lesions <2 cm diameter (Bryan *et al.*, 1977). CAT scanning, although apparently more sensitive than these 2 methods still appears to have a limitation in detecting small lesions in the liver (Scherer *et al.*, 1978). However, since it has been established that intrahepatic primary or secondary tumours are associated with an increased hepatic arterial blood flow (Breedis & Young, 1953) a study of the portal and arterial components of liver blood flow may represent a sensitive method of detecting metastatic involvement. We have initiated a prospective study on a group of patients with known gastrointestinal cancer. Dynamic flow scintigraphy (Sarper *et al.*, 1981) and static isotope scans were carried out prior to surgery, the results of these tests being correlated with the presence of hepatic metastases at laparotomy. In addition, flow scintigraphy was carried out on a group of healthy volunteers in order to establish a normal range of hepatic arterial and total hepatic blood flows.

Fifty nine patients with various types of gastrointestinal cancer were studied. Twenty four patients had colon cancer, 20 had rectal cancer and 15, gastric cancer. Of these, 25 were found to have hepatic metastases at laparotomy, and 34 had no obvious hepatic involvement. The control group comprised 20 healthy volunteers who underwent dynamic imaging.

After fasting for 12 h, subjects were positioned supine over a large field of view gamma camera in order to visualise and count over the liver, spleen, kidneys and lung bases. Following a rapid i.v. injection of 3 mCi (111 MBq) of Technetium-$99^m$-labelled tin colloid, image data were recorded in 2-second frames for 60 sec using a min-computer. After a further 15 min static liver images of at least $5 \times 10^5$ counts were acquired in anterior, posterior, lateral and oblique positions. From the stored dynamic data, regions of interest were selected corresponding to the right kidney and to the right lobe of liver (carefully excluding the lung bases) and time-activity curves generated. The time of the peak of the kidney curve was used to indicate the division between arterial and portal inflow phases of the liver curve. The quality of the bolus injection and its distribution was assessed by examination of the rise time of the kidney curve and studies were rejected if this was greater than 8 sec. After 3-point smoothing of the liver curve the average slopes of the 2 consecutive 8-second sections on either side of the arterial/portal division were calculated. The first slope was taken to represent arterial inflow and the second slope was taken to represent the portal inflow. The hepatic perfusion index (HPI) was expressed as a fraction of the arterial inflow to the total hepatic inflow. Static scans were independently assessed (P.J.R.) as being indicative or non-indicative of the presence of hepatic metastases. The results of the static and dynamic studies were correlated with findings at laparotomy for the presence or absence of hepatic metastases, which where possible were measured. The sites of these metastases were also noted.

Figure 1 shows the distribution of HPI values in the positive laparotomy group, in the group of patients with no liver metastases and in the control group. It can be seen that 24/25 patients who were in the laparotomy positive group (96%) had HPI values above the normal range, the upper limit of normal in this series being 0.42. One patient with massive hepatic replacement by tumour had an HPI value of 0.15 but interestingly had a positive static scan. In those patients known to have hepatic involvement, the sensitivity of static scanning was 64%. Nine patients in this group had normal scans, but all had abnormal HPI values. The data for the negative laparotomy group are also shown but no definitive statement can be made until the follow-up

Correspondence: S.H. Leveson, Department of Surgery, Level O8, Clinical Sciences Building, St. James's University Hospital, Leeds LS9 7TF.

Received 12 November 1982; accepted 26 January 1983.

© The Macmillan Press Ltd., 1983

**Figure 1** Scattergram showing the distribution of HPI values in the control group and in patients with and without overt hepatic metastases. Closed circles signify normal static scans; open circles signify abnormal static scans.

period is completed. It is evident, however, that in this group of 34 patients, 16 had HPI values above the normal range, and of these 2 had positive static scans. All patients in whom both HPI and laparotomy were negative had normal static scans.

Liver lesions <2 cm diameter are usually undetectable by radionuclide imaging and the proportion of patients in whom the deposits are <2 cm may be as high as 30% (Ozarda & Pickren, 1962). It is not surprising therefore that conventional scintigraphy underestimates the incidence of metastatic liver disease. Clearly a simple modification of routine liver scintigraphy which would allow the detection of lesions <2 cm diameter could be regarded as a clinically useful improvement. Although both ultrasound and CT scans have better spatial resolution than isotope images comparative studies have not shown a consistent improvement in sensitivity with these techniques (MacCarty *et al.*, 1979; Biello *et al.*, 1978; Scherer *et al.*, 1979). Sarper *et al.* (1981), using the recently developed technique of flow scintigraphy, have claimed a 100% sensitivity rate in detecting hepatic metastases, although in their series no rigorous clinical correlates were made. Using dynamic imaging we have been able to improve the sensitivity of the isotope scan by 50%.

Of considerable interest amongst this positive laparotomy group were those patients who had metastases <2 cm diameter which were often remote from the region of interest selected for the liver blood flow measurement. This would suggest the presence of either disseminated micrometastases or that even isolated small metastases within the liver may produce a soluble substance stimulating hepatic arterial blood flow. Using the procedure described above 96% of all patients with metastatic disease at laparotomy had perfusion indices >0.42. About one half of patients in whom the liver appeared normal at laparotomy also had arterial indices >0.42, giving a high 'false positive' rate using laparotomy as the final indicator. The 'true' incidence of false positive cases will more accurately be assessed after a period of follow-up since it is known that about half of occult metastases in patients with large bowel cancer declare themselves within a year of initial surgery (Olson *et al.*, 1980). The incidence of occult metastases in patients with primary large bowel malignancy is difficult to assess but in one recent series 11/43 patients had normal livers at laparotomy but presented with metastatic disease within 2 years (Finlay *et al.*, 1982). Whether the 'false positive' group in the present study includes patients with occult disease will be determined by follow-up examinations.

### References

BIELLO, D.R., LEVITT, R.G., SIEGEL, B.A. & 6 others. (1978). Computed tomography and radionuclide imaging of the liver: A comparative evaluation. *Radiology*, **127**, 159.

BREEDIS, C. & YOUNG, G. (1953). The blood supply of neoplasms in the liver. *Am. J. Pathol.*, **30**, 227.

BRYAN, P.J., DINN, M.W., GROSSMAN, Z.D., WISTOW, B.W., MCAFEE, J.G. & KIEFFER, S.A. (1977). Correlation of computed tomography, gray scale ultrasonography, and radionucleide imaging of the liver in detecting space-occupying processes. *Radiology*, **124**, 387.

FINLAY, I.G., MEEK, D.R., DUNCAN, J.G. & MCARDLE, C.S. (1982). Incidence and detection of occult hepatic metastases in colorectal cancer. *Br. Med. J.*, **284**, 803.

MACCARTY, R.L., STEPHENS, D.H., HATTERY, R. & SHEEDY, P.F. (1979). Hepatic imaging by computed tomography. A comparison with $^{99m}$TC-Sulfur Colloid, ultrasonography and angiography. *Radiol. Clin. N. Am.*, **17**, 137.

OLSON, R.M., PERENCEVICH, N.P., MALCOM, A.W., CHAFFEY, J.T. & WILSON, R.E. (1980). Patterns of recurrence following curative resection of adenocarcinoma of the colon and rectum. *Cancer*, **45**, 2969.

OZARDA, A. & PICKREN, J. (1962). The topographic distribution of liver metastases. Its relation to surgical and isotope diagnosis. *J. Nucl. Med.*, **3**, 149.

SARPER, R., FAJMAN, W.A., TARCAN, Y.A. & NIXON, D.W. (1981). Enhanced detection of metastatic liver disease by computerized flow scintigrams. *J. Nucl. Med.*, **22**, 318.

SCHERER, U., ROTHE, R., EISENBERG, & 10 others. (1978). Diagnostic accuracy of CT in circumscript liver disease. *Am. J. Roentgenol.*, **130**, 711.

SCHERER, U., SANTOS, M. & LISSNER, J. (1979). CT studies of the liver *in vitro*: A report on 82 cases with pathological correlation. *J. Comput. Assist. Tomogr.*, **5**, 589.